# Temporal degradation of data limits biodiversity research

**DOI:** 10.1002/ece3.3259

**Published:** 2017-07-27

**Authors:** Geiziane Tessarolo, Richard Ladle, Thiago Rangel, Joaquin Hortal

**Affiliations:** ^1^ Departamento de Ecologia Instituto de Ciências Biológicas Universidade Federal de Goiás Goiânia Brazil; ^2^ Programa de Pós‐graduação em Recursos Naturais do Cerrado Universidade Estadual de Goiás Anápolis Brazil; ^3^ ICBS Universidade Federal de Alagoas Maceió Brazil; ^4^ School of Geography and the Environment University of Oxford Oxford UK; ^5^ Departamento de Biogeografía y Cambio Global Museo Nacional de Ciencias Naturales (MNCN‐CSIC) Madrid Spain

**Keywords:** biodiversity data, data degradation, data quality, maps of ignorance, species distribution models, temporal decay

## Abstract

Spatial and/or temporal biases in biodiversity data can directly influence the utility, comparability, and reliability of ecological and evolutionary studies. While the effects of biased spatial coverage of biodiversity data are relatively well known, temporal variation in data quality (i.e., the congruence between recorded and actual information) has received much less attention. Here, we develop a conceptual framework for understanding the influence of time on biodiversity data quality based on three main processes: (1) the natural dynamics of ecological systems—such as species turnover or local extinction; (2) periodic taxonomic revisions, and; (3) the loss of physical and metadata due to inefficient curation, accidents, or funding shortfalls. Temporal decay in data quality driven by these three processes has fundamental consequences for the usage and comparability of data collected in different time periods. Data decay can be partly ameliorated by adopting standard protocols for generation, storage, and sharing data and metadata. However, some data degradation is unavoidable due to natural variations in ecological systems. Consequently, changes in biodiversity data quality over time need be carefully assessed and, if possible, taken into account when analyzing aging datasets.

## 1 INTRODUCTION

The quality of biodiversity data—that is, the degree of congruency between recorded data and current conditions that the historical data represents—is a central issue for global monitoring and assessment. It influences the accuracy of our descriptions of historical and contemporary patterns (Anderson, 2012; Goldewijk & Ramankutty, 2004), determining our ability to provide realistic models of the future impacts of environmental change (Hortal, Lobo, & Jiménez‐Valverde, 2012; Rocchini et al., 2011). Consequently, controlling for biological data quality is becoming increasingly important as advances in information technology promote ever faster gathering and access to biodiversity information (Chapman, 2005; Soberón & Peterson, 2004). Many museums, herbaria, and research centers now make their data available through centralized databases which can be publically accessed. The information contained within these databases often dates back to the beginnings of modern ecology (Magurran et al., 2010). While such data clearly have value for studying temporal changes in natural systems (Dornelas et al., 2013; Johnson et al., 2011; Magurran et al., 2010), if used uncritically it could generate serious biases and misunderstandings about contemporary biodiversity patterns and the processes that are responsible for them.

Existing research on biodiversity data quality has typically focused spatial bias, measurement errors, and sampling effort (Anderson et al., 2016; Boakes et al., 2010; Costello, Michener, Gahegan, Zhang, & Bourne, 2013; Hortal, Jimenez‐Valverde, Gómez, Lobo, & Baselga, 2008; Hortal et al., 2015; Rocchini et al., 2011; Soberón & Peterson, 2004; Veiga, Cartolano, & Saraiva, 2014). Indeed, the effects of data incompleteness and spatial bias have been long recognized (e.g., Colwell & Coddington, 1994; Margules & Austin, 1994; Soberon, Llorente, & Benitez, 1996) and recent developments have provided robust ways of describing these biases (see, e.g., McInerny et al., 2014; Meyer, 2016; Meyer, Weigelt, & Kreft, 2016; Ruete, 2015; Stropp et al., 2016) and, potentially, accounting for them in estimates of global change impacts on biodiversity (Anderson, 2013; Beale & Lennon, 2012; Hortal & Lobo, 2011; Ladle & Hortal, 2013; Rocchini et al., 2011).

The potential loss of quality of biodiversity information over time has received far less attention from researchers, possibly because the contemporary nature of many biodiversity data sets. Temporal degradation of biodiversity data quality is inevitable due to the inherent dynamism of natural systems (e.g., local extinctions, immigration, biological invasions). The dynamics drive—sometimes dramatic—temporal changes in the abundance and composition of species in ecological communities (e.g., Spitzer, Novotny, Tonner, & Leps, 1993; Holmes & Sherry, 2001; Forister et al., 2010; Dornelas et al., 2013; but see Vellend et al., 2013). Biodiversity data quality may also degrade due to changes in the way scientists divide and categorize biodiversity, exemplified by sporadic revisions (splitting and lumping) of taxonomic relationships (Ladle & Hortal, 2013). Finally, physical evidence such as voucher specimens and the associated metadata can be compromised due to inefficient curation, accidents, or the loss of funding (Chapman, 2005; Otegui, Ariño, Chavan, & Gaiji, 2013). All these issues produce a decrease of the congruency between values stored in a database and the ecological reality when the data are used. Such temporal biases have the potential to hamper descriptions of temporal variations in biodiversity and therefore limit our ability to model the effects of different stressors, incorporate them into estimates of global change impacts, and assess the outcomes of dynamic models through hindcasting.

The influence of time on data degradation is well‐known in other scientific fields and has been successfully incorporated into protocols to assess data quality (Kennedy et al., 2014; Peuquet, 1999; Veregin, 1999). In contrast, temporal degradation of biodiversity data is poorly understood and managed. This is despite many of our databases contain a high proportion of old records. The scale of this problem is well illustrated by the global analysis of Meyer et al. (2016), who observed that 62% of the 110 × 110 km grid cells with information on plant species at GBIF had no record after 1970. Similarly, in their analysis of GBIF data on African flowering plants, Stropp et al. (2016) found that the majority of well surveyed cells contained predominantly old records and were in urgent need of re‐sampling.

Another strong argument for explicitly dealing with degradation of biodiversity data is that temporal changes in the distribution of species and the composition of local assemblages can be large (Dornelas et al., 2013). For example, Escribano, Ariño, and Galicia (2016) estimated, based on land use images, that 75% of small mammal records in Spain were obsolete because they had been collected before or during land‐use changes. Significantly, rare species showed a greater rate of record obsoleteness constraining the use of these data for conservation planning. Likewise, data degradation due to taxonomic changes can also be large. For example, Hjarding, Tolley, and Burgess (2015) found that 99.9% of GBIF data about 35 chameleon species from Eastern Africa was taxonomically outdated, leading to 10 species being classified with an inappropriate threat status.

Clearly, the importance and magnitude of temporal degradation of data depends on the type of data and its current and future use. For studies that measure changes in biodiversity over time, historical data are essential. In these cases, the longer the temporal coverage, the better the study is able to capture the patterns of change. However, these changes are specially particularly limiting for temporally static researches. Such studies typically assume that biodiversity features and characteristics are temporally static with potential consequences for the accuracy of their inferences.

Here, we provide a conceptual framework for understanding the decay of biodiversity information over time based on three main processes: (1) the natural dynamics of ecological systems such as species turnover or local extinction; (2) taxonomic revision, and; (3) the loss of physical and metadata due to inefficient curation, accidents, or funding shortfalls. We then discuss the possible consequences of these processes on the temporal degradation of biodiversity data and suggest actions and policies to reduce and/or account for their effects.

## 2 A CONCEPTUAL FRAMEWORK FOR BIODIVERSITY INFORMATION DEGRADATION

**Figure 1:**
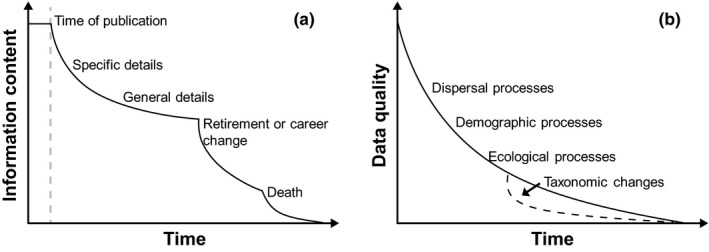
Temporal decay in the information on the current status of biodiversity that is provided by biodiversity data. (a) Data from field surveys start to loss ancillary information from the moment the samples are taken, or at least since the moment of publication (see Michener et al., 1997). As natural changes occur in the surveyed habitats, the access to ancillary information about the surveys starts to decay, through the loss of first specific and later general details on the surveys. This information becomes progressively less accessible when the researchers involved in the surveys retire or take a career shift and are definitively lost with their death. (b) The quality of biological records to provide an accurate picture of the current status of biodiversity decays with time as assemblage composition and species distributions change with time due to natural processes (i.e., dispersal, demographic, and other ecological processes), a process that is aggravated by changes in the taxonomy of the studied groups, that hamper matching old records with currently recognized species accurately

Ecological data typically degrade following characteristic phases that can be conceptualized in terms of information entropy (Figure 1a) (Michener, Brunt, Helly, Kirchner, & Stafford, 1997). Loss of data quality begins immediately after data collection, with specific details disappearing first, followed by loss of more general information. In the case of species‐level biodiversity data, these changes are promoted by a complex array of natural, technical, and societal changes, including environmental change, the improvement in taxonomic tools and taxonomic knowledge, and the loss of part of the ancillary information (e.g., location or date) associated with the records.

The most rapid temporal changes in biodiversity data quality are typically caused by changes in the sampled environment and begin as the moment sampling is complete (Ladle & Hortal, 2013). These environmental changes may be natural variations or caused by human interference (e.g., habitat loss or degradation and are enacted through local extinction or immigration events (Figure 1b). An extreme example would be data about the presence (or absence) of species in an area of rainforest that has been transformed into a sugar cane plantation. Although a few forest‐dependent species may still occur within the original sample area in isolated fragments, many species will be locally extinct or already undergoing local extinction. Conversely, a few new generalist or matrix tolerant species may have immigrated into the area. Under such a scenario, using the original data to train species distribution models intended to represent current distributional ranges would generate unrealistic results.

Of course, ecosystems do not need to be completely transformed to cause temporal decay in the quality of biodiversity data. The constantly changing nature of species assemblages, whereby some species emigrate and new species arrive, is sufficient to cause information loss over time. Recent studies demonstrate that considerable change in assemblage composition can occur over a very short space of time (Buckley, 2013; Diekmann et al., 2014; Parody, Cuthbert, & Decker, 2001). Species turnover occurs naturally due to demographic, environmental, and population stochasticity or may be driven by dynamic biophysical processes including climate change or alterations in habitat structure due to anthropogenic pressures. Such changes influence the suitability of habitats for numerous species, driving further bouts of local extinction and/or immigration. The net effect of these biophysical modifications is that community composition and, therefore, species distributions vary over time.

Even if the assemblage within the sampled area remains more or less unchanged, the quality of the information may still degrade due to the behavior of scientists. For example, the frequent career changes of field collectors and museum curators may prompt the loss of valuable ancillary information gathered during field campaigns (Figure 1a). Good‐quality metadata is not only necessary to understand the data, but to effectively assess its quality, context, content, and accessibility. Such information is essential for contemporary scientists to assess and use older data (Costello et al., 2013). Therefore, the loss of the information associated to the specimens held in natural history collections also increases the rate of degradation of data quality with time, in particular when the original field notes are not properly stored and/or detailed information on the sampling event is not recorded in the voucher labels. Here, a careful curation of the metadata when digitizing the collections and sharing with biodiversity information networks is key to safeguard as much as possible of the information stored in biological records (see Chapman, 2005).

Changes in the taxonomic status of some species due to reclassification can also reduce the accuracy of species lists (Hey, Waples, Arnold, Butlin, & Harrison, 2003; Isaac, Mallet, & Mace, 2004). Reclassifications include merging several species into a single one (“lumping”), or the division of one species into two or more species (“splitting”). Both lumping and splitting can influence estimates of local diversity, conservation status, and the geographic distribution of species (Isaac et al., 2004). However, while accounting for lumping is relatively easy using database updates, correcting for splitting requires review of the original voucher specimens (if they still exist—see above) to assign each record to one of the new taxa.

Voucher specimens thus play a key role in maintaining biodiversity data quality, in respect to data degradation due to both loss of metadata and/or taxonomic changes. If data are associated with identifiable vouchers that have been stored and included in collections, it can often be revised in light of taxonomic changes that took place after the original study was published. This is also true for digitally stored information, such as recordings of bird songs/bat calls or photographs. Conversely, if the only information is a species name on a list, then the only remedial measures that can be adopted are in cases of lumping species.

Although the degrading influence of time on biodiversity data quality has been recognized (Magurran et al., 2010; Stropp et al., 2016), little progress has been made in the development of strategies to manage the consequences of such temporal degradation. Indeed, it is common practice to treat biogeographical data collected at different times as being equivalent. For example, in Species Distribution Modelling, occurrence data are commonly used without accounting for collection dates or the time when specimens were taxonomically identified. SDMs are constructed on the assumption that species distributions are mainly driven by climatic factors, and this relationship is derived from the climate variables at the postulated site of occurrence. Such models implicitly adopt a static view of niches (but see Soberón & Nakamura, 2009), making the assumption that areas with suitable environmental conditions will remain the same over time. This is clearly unrealistic, given that many studies have documented changes in the realized niche can occur over relatively short time periods, particularly during biological invasions (Broennimann et al., 2007; Rödder & Lötters, 2009; Silva, Vilela, Buzatto, Moczek, & Hortal, 2016). Thus, distributions based on old occurrence data and their associated (contemporary) climate characteristics are unlikely to be representative of the current species niche (Boitani et al., 2011). The robustness of such models could potentially be improved by accounting for the influence of time on the quality of occurrence data. Such an approach would also benefit the increasing number of macroecological studies based on big data that do not assess the temporal consistency of the data they use. These data often come from checklists or range maps developed from occurrence data typically gathered across several decades (Hortal, 2008).

## 3 DEALING WITH DEGRADATION: ACTIONS AND POLICIES

Loss of biodiversity data quality over time is unavoidable, although certain actions can be taken to slow the rate of degradation. With respect to taxonomic revisions, data should be updated to take into account the latest taxonomic changes. Failure to follow this simple strategy can lead to misleading patterns of biodiversity and associated conservation prioritization strategies. Here, we present some solutions to address these issues using examples from both biodiversity research and from other scientific areas (summarized in Table 1).

**Table 1:** Solutions to overcome the temporal degradation of biodiversity data

Problems	Solutions	Examples	References
Misleading and outdated taxonomy	Periodically update databases	Data can be checked using Taxonomic Authority Files (TAFs), that are reference lists of taxonomic names. If TAFs are not available for the studied group, a careful checking of the taxonomic literature is needed.	Vanden‐Berghe et al. (2015), Zermoglio et al. (2016), Nguyen et al. (2017)
	Increase access to voucher specimens	Posting information about curators and policies of access vouchers in online databases can help researches to find relevant material coming from different origins that is sparse in different collections. In addition, the digitalization of vouchers in high‐resolution images and its storage in online databases can make many collections available to researches and general public almost instantaneously.	Beaman & Cellinese (2012), Gries et al. (2014), Skevakis et al. (2014), Schindel et al. (2016), Ellwood et al. (2015)
Degradation of metadata	Follow standard protocols to record metadata	Protocols that indicate which information should be recorded, and how, are available for different areas. Following them can facilitate sharing the metadata in online databases.	Michener et al. (1997), Fegraus et al. (2005), Chapman (2005), Wieczorek et al. (2012)
	Publish all kinds of metadata related to the project	Metadata can be published together with project results, as part of the supplementary materials at the same journal. Such behavior can be encouraged if journals require and peer review such metadata during the review process of the submitted papers. Also, metadata can be shared and archived in online platforms designed for such objective.	Costello et al. (2013), Foster & Deardorff (2017)
	Deposit and curate voucher specimens	Physical vouchers should be deposited in permanently and publicly accessible repositories. Concomitantly, it is necessary to increase in the funds for curation and collection management.	Turney et al. (2015), McLean et al. (2016)
Natural and unnatural variations	Downweight or remove data considered obsolete	Older data can be downweighted, so that they contribute less to final results. Curves of temporal decay in the relevance of information can be generated, based the on factors leading to information degradation. Also, older data can be removed from analysis (but see our recommendations throughout the text).	Boitani et al. (2011), Yu & Placide (2012), Giraitis et al. (2013), Viele et al. (2014), Meyer et al. (2015), Stropp et al. (2016)
	Incorporate temporal degradation and uncertainty in analysis	Analysis can be improved by the incorporation of uncertainty about temporal degradation information using weights as covariates in the modeling process. Degradation can also be added as a stochastic component to assess the sensitivity of the results to such variation.	Hordijk & Broennimann (2012), Rocchini et al. (2011), Stropp et al. (2016)
	Assess obsolete information and identify areas to carry out new survey campaigns	New surveys should be planned focusing on sites in which surveys will generate more updated information. Surveys can be planned accounting for those sites that hold the oldest records or by identifying areas that suffered significant land use and/or environmental changes after the last collection.	Stropp et al. (2016), Escribano et al. (2016)

Managing misleading and outdated taxonomy in biodiversity databases is technically and logistically challenging. However, recent initiatives to create definitive reference lists of taxonomic names have rendered such a strategy feasible. For example, Taxonomic Authority Files (TAFs; Vanden‐Berghe et al., 2015; Zermoglio, Guralnick, & Wieczorek, 2016) are now available for a wide range of spatial and taxonomic scales, including catalogue of life (http://www.catalogueoflife.org), Integrated Taxonomic Information System (http://www.itis.gov), Mammal Species of the World (Wilson & Reeder, 2005), The Plant List (http://www.theplantlist.org/), or FishBase (Froese & Pauly, 2017). TAFs can be used to assess and update biodiversity data using currently accepted taxonomic names. Although methods for automated taxonomic checking in TAFs are still under development (Nguyen, Soto, Kontonatsios, Batista‐Navarro, & Ananiadou, 2017; Vanden‐Berghe et al., 2015), manual taxonomic checking can be carried out for small databases or for subsets of data (Zermoglio et al., 2016).

Degradation of metadata is more straightforward to deal with. Such associated or derived data are essential to maintain the quality and, consequently, the usability of biodiversity data over time (Costello et al., 2013; Huettmann, 2009). There have been repeated calls over the last few years for publishing metadata along with project data in an attempt to increase the longevity of the latter (Costello & Wieczorek, 2014; Michener, 2015). To facilitate this, protocols for documenting and publishing metadata associated to biodiversity data have been developed (Fegraus, Andelman, Jones, & Schildhauer, 2005; Michener et al., 1997; Wieczorek et al., 2012). Unfortunately, the use of such protocols is not widespread and many datasets still contain a considerable number of errors (Kervin, Michener, & Cook, 2013; Tenopir et al., 2011). Further action is clearly required and, recognizing this, some scientific journals now oblige researchers to provide both their data and metadata for peer review and publication (Costello et al., 2013). Likewise, data management tools such as those provided by the Open Science Framework (OSF; https://osf.io/) provide platforms for sharing and archiving metadata and analysis, thereby increasing the lifespan of information and facilitating replication of collection methods (Foster & Deardorff, 2017).

Another way to improve the quality and usability of biodiversity data over time is through careful curation and management of vouchers specimens (Costello & Wieczorek, 2014). There are an increasing number of initiatives that aim to connect physical specimen information to citations and other kinds of records (Schindel, Miller, Trizna, Graham, & Crane, 2016; Skevakis, Makris, Kalokyri, Arapi, & Christodoulakis, 2014). Such information can be used to identify the institutions that originally held the specimen and to track possible changes of institution. Additionally, entire collections are being digitalized and posted to online databases, allowing researchers to gain direct access to high‐resolution images (Beaman & Cellinese, 2012; Ellwood et al., 2015; Gries, Gilbert, & Franz, 2014). Such digitalization, while useful, does not supersede the maintenance of physical vouchers which are still essential for the majority of taxonomic re‐evaluations (Balke et al., 2013; Culley, 2013). Perhaps surprisingly, given their importance for maintaining biodiversity data quality, depositing vouchers (specimens, photography, DNA) in permanently and publicly accessible repositories is not normative behavior among the majority of researchers (McLean et al., 2016; Turney, Cameron, Cloutier, & Buddle, 2015).

A related issue is the difficulty of merging and/or comparing historical and contemporary data due to changes in methodology or sampling biases over time. Generally, older samples were collected with nonstandard and now outdated methodologies, making them more prone to inaccuracies (Tingley & Beissinger, 2009). These inaccuracies can have significant consequences for studies trying to detect biodiversity changes over time, as the baseline data may be biased leading to misleading inferences about the extent and rate of environmental change (Knutson et al., 2010; Skelly, Yurewicz, Werner, & Relyea, 2003). For example, the objectives of historical collecting expeditions were often strongly influenced by economic imperatives or the preferences of private funders. Conversely, new technologies such as camera traps are capable of detecting the presence of previously elusive species (Ladle, Jepson, Malhado, Jennings, & Barua, 2011). Thus, to allow comparability, historical data should be assessed for under‐ or over‐detected events in relation to contemporary data (Knutson et al., 2010). These can then be removed or taken into account in the analyses. For example, Moritz et al. (2008) estimated rates of change in small‐mammal communities by controlling for differences in detectability of species in historical and current surveys. Using a similar approach, Tingley and Beissinger (2009) propose the use of occupancy models to remove bias in historical data to correctly detect species range shifts.

The influence of natural (or unnatural) environmental changes on biodiversity data cannot be remediated, but can be taken into account. Perhaps the simplest approach is to assign weights to each occurrence record in relation to when it was collected, adjusting the data to specific curves representing the rate of information decay. This approach is widely used in other fields that work with temporally variant data (Giraitis, Kapetanios, & Price, 2013; Viele et al., 2014; Yu & Placide, 2012). Data weights could be arbitrarily defined by the user (as in, e.g., Meyer, Kreft, Guralnick, & Jetz, 2015 or Stropp et al., 2016). But, alternatively, here we propose that these weights could be generated through curves of temporal decay in information quality. The slope of these curves would be dependent on the main factors causing the degradation of biodiversity data, such as (1) the reliability of taxonomic identifications—taken from, for example, the temporal variation in the rate between valid names and synonyms (Baselga, Hortal, Jiménez‐Valverde, Gómez, & Lobo, 2006); or (2) mean species turnover rates—taken from, for example, species–time relationships (White et al., 2006). A more extreme approach would be to remove data considered as outdated (Boitani et al., 2011). However, we would argue that weighting is a better approach as it may allow researchers to address important questions about environmental change while remaining cognizant of the potential biases inherent in historical data. Moreover, as there are currently no quantitative studies about the life span of different forms of biodiversity data and their uses, any arbitrary threshold to remove data would have the effect of reducing data quality by introducing other uncertainties (Meyer et al., 2016).

More generally, the temporal and spatial decay of biodiversity information should be formally and systematically incorporated into modeling and conservation planning decisions (Ladle & Hortal, 2013). This could practically be achieved by adding weights as covariates during the modeling process (Beale & Lennon, 2012) or through data management procedures. A good example of the latter is the study of Yu and Placide (2012), in which they used a decision tree technique to build an information decay‐based predictive model. For biodiversity analysis, the incorporation of temporal data decay could be achieved through the development of “Maps of Ignorance” (proposed by Rocchini et al., 2011); that is, geographically explicit representations of uncertainty generated by assigning weights to each occurrence record to create spatial representation of the reliability of data. Although the proposed “Maps of Ignorance” consider other sources of uncertainty in data (such as geographical and climatic bias), they could also explicitly address the temporal data quality issues (see Figure 4 in Stropp et al., 2016) and, potentially, be used as a source of data for a covariate matrix during modeling.

Another promising way to deal with uncertain data is by taking into account the variation that the stochasticity of an event generates on the intended analysis. For example, Hordijk and Broennimann (2012) used a stochastic approach to deal with the uncertainty of the time of first occurrence observations when developing a new method to reconstruct dispersal routes. The authors added a stochastic component to their analysis by subtracting from each record a random value derived from a distribution that simulated the time that an observer would need to discover a plant by chance. In order to estimate the uncertainty of these events, they repeated the simulation 100 times, thereby assessing the robustness of the results in face of stochastic variations.

Although the solutions mentioned above may slow the loss of data quality, they cannot prevent data from continuing to degrade over time. Thus, without new surveys, a biodiversity database will inevitably become less useful. Approaches such those applied by Stropp et al. (2016) and Escribano et al. (2016), can be used to prioritize species for new sampling campaigns and identify areas where knowledge is primarily from old records.

## 4 CONCLUDING REMARKS

The passage of time imposes unavoidable limits on the sustained usefulness of information about the natural world (Bergstrom, 2012). However, the temporal decay of data quality influences different types of studies in different ways and its impact should therefore be evaluated on a case‐by‐case basis. Temporal information decay of species data can affect perceived geographic distribution patterns and associated strategies of conservation prioritization (Hortal et al., 2015). Moreover, the decay of biodiversity information quality with time cannot be completely mitigated and efforts should therefore focus on developing tools to manage it.

Finally, it is important to remember that we (authors, editors and reviewers) are jointly responsible for the quality of the data used in publications and should therefore be aware of both the limitations of older data and the need to prolong the useful life span of the data being collected now (Costello et al., 2013). Specifically, authors should clearly state whether, and how, the passage of time may affect the strength of their inferences. Likewise, editors and reviewers should carefully check that articles that utilize biodiversity data have appropriately addressed temporal information decay.

## CONFLICT OF INTEREST

None declared.

## AUTHOR CONTRIBUTIONS

The ideas of the manuscript were conceived and discussed by all authors. G.T. wrote the manuscript, and R.L., T.F.R, and J.H commented and edited the manuscript.
